# Combined analysis of the triglyceride–glucose index and melanin-concentrating hormone in metabolic dysfunction–associated fatty liver disease: a machine learning–based study

**DOI:** 10.3389/fnut.2026.1763190

**Published:** 2026-03-05

**Authors:** Xiuyuan Hong, Ling Li, Qi Huang, Xiaoying Yuan, Ying Zhang, Han Zhang, Qingqing Wang, Yan Deng, Dingyan Luo, Yue Yuan, Qi Zeng, Xin Liao

**Affiliations:** 1 Department of Endocrinology and Metabolism, The Affiliated Hospital of Zunyi Medical University, Zunyi, China; 2 Department of Clinical Medicine, Zunyi Medical and Pharmaceutical College, Zunyi, China; 3 Department of Nuclear Medicine, The Affiliated Hospital of Zunyi Medical University, Zunyi, China; 4 Department of Endocrinology and Metabolism, Honghuagang District People’s Hospital, Zunyi, China; 5 Guizhou Provincial Key Laboratory of Pathogenesis and Prevention of Common Chronic Diseases, Guizhou, China

**Keywords:** metabolic dysfunction-associated fatty liver disease, melanin-concentrating hormone, triglyceride-glucose index, machine learning, prediction model

## Abstract

**Objective:**

Metabolic dysfunction-associated fatty liver disease (MAFLD), a highly prevalent global liver disorder, requires simple and accessible screening approaches. As current diagnostic methods, such as the Controlled Attenuation Parameter (CAP), are limited in their applicability in obese patients and are primarily designed for fibrosis assessment. This study aim to investigate the associations of the serum melanin-concentrating hormone (MCH) and triglyceride–glucose (TyG) indices with MAFLD and to explore the risk factors and disease probability of MAFLD by developing machine learning models.

**Methods:**

In this cross-sectional study of 212 MAFLD patients and 107 healthy controls, and feature selection were identified through the least absolute shrinkage and selection operator (LASSO) regression analysis and Variance Inflation Factor (VIF). Three predictive models—Logistic Regression, Random Forest, Support Vector Machinemodel (SVM)—were constructed using the training set and evaluated in an independent test set. Construction of nomogram using independent risk factors screened by machine learning. Multivariate logistic regression analysis was used to explore further assess independent risk factors. Mediation analysis was conducted to explore potential pathways.

**Results:**

Logistic regression model was found to outperform other classifier models in testing data [area under the curve (AUC) of 92.6, 95% CI: 0.865–0.987] and achieve the lowest Brier Score as well. Decision curve analysis suggested potential clinical utility. Logistic regression analysis indicated that MCH (OR, 2.193; 95% CI, 1.242–3.873; *P* = 0.007), TyG index (OR, 1.002; 95% CI, 1.001–1.003; *P* < 0.001), are independent risk factors for MAFLD. Subgroup analysis of the association between MCH and MAFLD stratified by sex, age, and body mass index (BMI) showed no significant effect modification after adjustment. Mediation analysis indicated that the TyG index accounted for a modest proportion of the association between MCH and MAFLD (mediation proportion: 10.89%).

**Conclusion:**

Serum MCH and the TyG index were independently associated with MAFLD. A machine learning–based screening model was developed and internally validated, showing promising performance for identifying individuals at higher risk. However, external validation in larger multicenter prospective cohorts is warranted before broader clinical application.

## Introduction

Metabolic dysfunction-associated fatty liver disease (MAFLD), formerly known as non-alcoholic fatty liver disease (NAFLD), has emerged as a significant global public health problem. As a key hepatic manifestation of metabolic syndrome, MAFLD affects approximately one -quarter of the global adult population, with its prevalence in China reaching as high as 27–30% (1). This disease is closely associated with obesity, insulin resistance (IR), and metabolic syndrome and serves as an independent risk factor for liver cirrhosis and hepatocellular carcinoma (2). With the increasing adoption of unhealthy lifestyles and the growing prevalence of obesity and diabetes, the incidence of MAFLD continues to rise. MAFLD is widely recognized as a major risk driver for cardiovascular diseases, kidney diseases, and type 2 diabetes (3–6). Given the critical role of hepatic steatosis in accelerating disease progression toward fibrosis and cirrhosis (7), early identification and diagnosis of MAFLD are crucial for improving clinical outcomes (8). However, the use of liver biopsy, the gold standard for diagnosis, is significantly limited for population screening because of its invasiveness, high cost, and potential complications. Although non-invasive techniques such as the controlled attenuation parameter (CAP) obtained from transient elastography (TE) provide a quantitative measure correlated with the histological grade of steatosis (9), their widespread applicability and accuracy in clinical practice remain challenging. A large-scale meta-analysis indicated that the diagnostic performance of CAP is significantly influenced by multiple factors, including BMI, diabetes status, and the stage of liver fibrosis, necessitating careful interpretation in the context of clinical findings (10). Furthermore, a large-scale prospective study reported a measurement failure rate as high as 7.7% and identified female sex, high BMI (> 30 kg/m^2^), and metabolic syndrome as independent risk factors for measurement failure (11). Therefore, identifying and validating convenient, cost-effective, and reliable non-invasive biomarkers has significant clinical value for early screening, risk stratification, and disease monitoring of MAFLD.

Against this backdrop, the triglyceride-glucose (TyG) index, a composite indicator calculated from serum triglyceride (TG) and fasting plasma glucose (FPG) levels, has emerged as a preferred surrogate marker for assessing IR (12). The TyG index can reflect the core pathological state of IR driven by both “glucotoxicity” and “lipotoxicity.” It has gained wide clinical acceptance because of its advantages, including simple calculation, low cost, and high sensitivity and specificity (13). IR and lipid metabolismare involved in the pathogenesis and progression of MAFLD (2). Numerous studies have confirmed a favorable association between the TyG index and various metabolic diseases, including MAFLD (14, 15). However, the TyG index essentially reflects a systemic IR status rather than liver-specific pathophysiological processes, which may limit its specificity for the precise prediction and risk assessment of MAFLDwhen used alone (16). Mammalian melanin-concentrating hormone (MCH) is a 19-amino-acids peptide (17). MCH-producing neurons are specifically located in the lateral hypothalamic area (LHA) and zona incerta and have extensive projections throughout the brain (18). MCH has a wide range of biological functions, including the regulation of sleep, emotion, memory, and reproduction (19, 20). After intracerebroventricular injection of MCH was demonstrate to induce feeding in rats, this hormone rapidly came to be recognized as pivotal in energy regulation and metabolic homeostasis (21). Moreover, MCH is not only regulates feeding behavior and energy homeostasis, but elevated MCH levels have also been shown to directly induce hyperglycemia and insulin resistance (22, 23). More importantly, preclinical studies have demonstrated that central administration of MCH can significantly induce hepatic steatosis, weight gain, and hepatic triglyceride accumulation in mice, possibly through a mechanism involving autonomic nervous system to promote fat deposition (24). These findings suggest that MCH plays a direct role in the pathophysiological network linking glucose and lipid metabolism disorders, IR, and hepatic fat accumulation. At present, existing studies on melanin-concentrating hormone (MCH), both domestically and internationally, have primarily focused on its roles in sleep regulation, appetite control, and energy homeostasis. Most of these studies are based on animal models and cellular experiments and have largely concentrated on the functions of central MCH. In contrast, evidence regarding the association between circulating MCH and MAFLD remainslimited. In recent years, machine learning models, with their strengths in big data analysis, artificial intelligence, and visualization, have been widely applied in disease prediction (25, 26). This study aimed to investigate the associations between circulating MCH, the TyG index, and MAFLD, and to develop machine learning–based screening models that may facilitate early risk stratification of MAFLD in clinical settings.

## Materials and methods

### Study participants

This study recruited 500 participants who underwent physical examinations and abdominal ultrasound scans at the health checkup center of the Affiliated Hospital of Zunyi Medical University between August 2021 and August 2022. The exclusion criteria were as follows: age under 18 years or over 75 years; incomplete data; clinical diagnosis of liver cirrhosis or hepatocellular carcinoma; history of malignant tumors; alcohol consumption > 20 g/day for men, > 10 g for women; history of viral hepatitis; pregnancy or lactation; and other endocrine disorders. Ultimately, 319 participants were included (Supplementary Figure 1, see Supplementary material associated with this article online).

The diagnosis of metabolic dysfunction-associated fatty liver disease (MAFLD) was established according to the 2020 international expert consensus criteria (1). The diagnosis requires evidence of hepatic steatosis in conjunction with at least one of the following three conditions: (1) overweight or obese, defined as a body mass index (BMI) ≥ 24 kg/m^2^ in Asians; (2) presence of type 2 diabetes mellitus (T2DM); or (3) evidence of metabolic dysregulation, defined as the presence of at least two of the following metabolic risk factors: waist circumference ≥ 102 cm in men or ≥ 88 cm in women; blood pressure ≥ 130/85 mmHg or receiving antihypertensive drug treatment; plasma triglycerides ≥ 150 mg/dL (1.70 mmoL/L) or receiving drug treatment; and plasma high-density lipoprotein cholesterol (HDL-C) < 40 mg/dL (1.0 mmoL/L) for men or < 50 mg/dL (1.3 mmoL/L) for women.

### Data collection and measurements

After general clinical data were from electronic medical records, venous blood samples were collected from all participants. Routine biochemical parameters, including liver function, renal function, and lipid profiles, were measured on a Beckman Coulter AU2700 automated analyzer (Beckman Coulter, Inc., Brea, CA, United States). Fasting plasma glucose (FPG) was determined via the hexokinase method via reagents from Beckman Coulter. Fasting insulin levels were quantified via a chemiluminescence immunoassay on a Beckman Coulter immunoassay system (Beckman Coulter, Inc., United States). For the measurement of melanin-concentrating hormone (MCH), whole blood samples were centrifuged at 3,000 rpm for 20 min. The resulting serum was carefully aspirated, aliquoted into 0.2 mL labeled cryovials, and immediately stored at –80°C until analysis. Serum MCH levels were measured with a commercial ELISA kit (Jiangsu SuMeiKe Biotechnology Co., Ltd.). The assay was performed according to the manufacturer’s protocol, and the concentrations were determined by measuring the optical density at 450 nm against a standard curve.

### Calculated metabolic indices

Several validated indices were calculated to assess metabolic status. HOMA-IR was calculated as follows: HOMA-IR = [FPG (mmol/L) × FINS (mU/mL)]/22.5. The triglyceride glucose (TyG) index was calculated using the following formula: TyG = ln [(TG (mg/dL) × FPG (mg/dL))/2] (27). BMI was calculated as weight (kg) divided by height (m)^2.

### Abdominal ultrasound assessment

All participants underwent fasting abdominal ultrasonography to evaluate hepatic steatosis. Examinations were performed by experienced sonographers who were blinded to the clinical and laboratory data. To minimize operator-dependent variability, standardized diagnostic criteria for fatty liver were applied across all assessments. The diagnosis of hepatic steatosis was based on characteristic ultrasonographic features, including enhanced hepatorenal echo contrast with distal attenuation, mild-to-moderate hepatomegaly with rounded edges, and decreased intrahepatic blood flow signals. Ultrasonographic examinations were conducted using a Siemens GE LOGIQ E8 ultrasound system with consistent device settings within the same outpatient imaging center, with routine quality control procedures in place.

### Delirium assessment

Feature Selection aims to discover the optimal feature set for constructing the model of interest. Effective feature selection can improve classification accuracy, whereas using too many features may lead to overfitting and be unsuitable for model construction. In this study In this study, two feature selection techniques were employed, such as Least absolute shrinkage and selection operator (LASSO) and Variance Inflation Factor (VIF), to enhance model performance.

### Machine learning models

In this study, three distinct machine learning models were employed for both training and testing, namely, Support Vector Machines (SVM), Logistic Regression (LR), Random Forest (RF). RF is an integrated learning method based on decision trees. It operates on the logic of improving the accuracy and robustness of the model by constructing multiple decision trees based on random samples and random features. This model is a powerful machine learning model and is a good choice for solving classification problems (28). SVM is a supervised machine learning algorithm that can be used for regression and classification problems. It functions by delineating data into decision boundaries for varied classes, concurrently maximizing the margin between these boundaries and the nearest data instances, thereby enhancing the model’s classification performance and generalization capability. LR is a generalized linear regression model which is commonly used to solve classification problems, this model is easy to understand and explain.

### Selection of machine learning models

The initial population was randomized into training and testing groups at a ratio of 8:2, which were subsequently utilized for model development and testing respectively. A five-fold cross-validation (five-fold CV) procedure was applied to the training dataset to internally validate the model. The training data were randomly partitioned into five approximately equal subsets (“folds”). In each iteration, four-folds were used to train the model and the remaining fold served as the validation fold. This process was repeated five times, with each fold used exactly once for validation. Internal five-fold cross-validation was employed to discern the most suitable hyperparameters for each distinct model, individually applied to each model for enhanced precision. Moreover, external five-fold cross-validation facilitated the comparison of machine learning models, identifying the model with superior average performance as the ultimate predictive model (the parameters of the four machine models are shown in Supplementary Table 1). In addition, bootstrap resampling with 1,000 iterations was performed to further assess the stability and robustness of the model. Subsequently, the model performance was further evaluated in an independent testing set. Discrimination and calibration were used to verify the predictive ability of the model. Discrimination was evaluated using the area under the receiver operating characteristic curve (AUROC). Model performance was further assessed using accuracy, sensitivity, specificity, and F1 score Calibration was evaluated using the Brier score and calibration curves. The Brier score represents the mean squared difference between the predicted probabilities and the observed outcomes, with lower values indicating better model performance (29). Decision curve analysis (DCA) was used to assess both discriminative ability and clinical utility.

### Statistical analysis

R software (version 4.5.2) and GraphPad Prism (version 9.1) were used to analyze the data in this study. Outliers were identified within the training dataset using the interquartile range (IQR) method, defined as values below Q1 - 1.5 × IQR or above Q3 + 1.5 × IQR. All identified outliers were subsequently reviewed against the original source records. Values attributable to data entry errors were corrected to their accurate values, whereas observations confirmed as true extreme values were retained and handled using winsorization by capping them at the corresponding IQR-based thresholds. If the missing value percentage is more significant than 20%, it will be excluded from the final completed dataset. If the rate of missing value is smaller than 20%, the multiple imputation by chained equations method would be used for imputation. Continuous variables were standardized using z-score normalization prior to model training to ensure comparability across predictors, particularly for algorithms sensitive to feature scaling, such as logistic regression and support vector machines (Supplementary Table 2, see Supplementary materials associated with this article online). After Shapiro–Wilk normality testing, the continuous variables were presented as the means ± SDs or medians [IQRs] and were compared via t tests or Mann–Whitney U tests, respectively. Categorical variables, presented as n(%), were compared using the χ^2^ test or Fisher’s exact test. Multivariate logistic regression was used to identify the independent risk factors for MAFLD in this study. Mediation analysis was performed, adjusting for relevant confounders, to further explore the interrelationships between variables. *P* < 0.05 were considered statistically significant.

## Results

### Baseline characteristics of the study population

A total of 319 participants (174 men and 145 women) were enrolled in this study, comprising 212 patients with metabolic dysfunction-associated fatty liver disease (MAFLD) and 107 individuals without MAFLD, who composed the non-MAFLD control group. The participant characteristics are summarized in Table 1. Compared with non-MAFLD patients, MAFLD patients were significantly older (45.65 vs. 41.58 years; *P* < 0.05), had a greater proportion of males, and had significantly greater BMIs; ALT, AST, MCH, TG, and HOMA-IR; and TyG indices. Conversely, their HDL-C levels were significantly lower (all *P* < 0.05). No significant differences were found in total cholesterol (TC) or LDL-C levels between the groups.

**TABLE 1:** Demographic and clinical characteristics of participants by the presence of MAFLD.

Variable	Total (*n* = 319)	NC (*n* = 107)	MAFLD (*n* = 212)	Statistic	*P*
Age, mean ± SD	44.28 ± 10.97	41.58 ± 10.74	45.65 ± 10.85	*t* = –3.172	0.002
Gender, n (%)				χ^2^ = 19.127	< 0.001
Male	174 (54.55)	40 (37.38)	134 (63.21)		
Female	145 (45.45)	67 (62.62)	78 (36.79)		
Height, mean ± SD	152.97 ± 40.96	161.57 ± 7.86	148.62 ± 49.41	*t* = 3.725	< 0.001
Weight, mean ± SD	67.35 ± 11.65	59.69 ± 9.64	71.21 ± 10.65	*t* = –9.413	< 0.001
BMI, mean ± SD	25.02 ± 3.09	22.83 ± 2.83	26.13 ± 2.59	*t* = –10.393	< 0.001
SBP, mean ± SD	127.21 ± 17.13	119.06 ± 15.35	131.32 ± 16.53	*t* = –6.405	< 0.001
DBP, mean ± SD	79.93 ± 12.49	73.77 ± 10.98	83.05 ± 12.06	*t* = –6.684	< 0.001
FPG mean ± SD	6.34 ± 2.82	4.88 ± 0.51	7.08 ± 3.20	*t* = –9.793	< 0.001
ASTALT, mean ± SD	1.11 ± 0.43	1.37 ± 0.46	0.97 ± 0.34	*t* = 7.901	< 0.001
TBil, mean ± SD	13.29 ± 6.22	13.30 ± 4.83	13.29 ± 6.81	*t* = 0.016	0.987
Cr, mean ± SD	76.48 ± 55.43	72.39 ± 19.27	78.55 ± 66.57	*t* = –0.936	0.350
TC, mean ± SD	5.51 ± 1.56	5.34 ± 1.12	5.60 ± 1.73	*t* = –1.363	0.174
HDL-C, mean ± SD	1.25 ± 0.31	1.45 ± 0.30	1.15 ± 0.26	*t* = 9.236	< 0.001
MCH, mean ± SD	11.56 ± 3.05	9.53 ± 2.74	12.58 ± 2.66	*t* = –9.592	< 0.001
TyG, mean ± SD	9.00 ± 0.77	8.47 ± 0.56	9.27 ± 0.72	*t* = –10.948	< 0.001
ALT, M (Q*1*, Q*3*)	26.00 (17.00, 41.00)	19.00 (14.00, 28.00)	31.00 (21.00, 45.00)	*Z* = –7.007	< 0.001
AST, M (Q*1*, Q*3*)	27.00 (22.00, 33.00)	25.00 (22.00, 28.00)	28.00 (23.00, 36.00)	*Z* = –3.495	< 0.001
TG, M (Q*1*, Q*3*)	1.81 (1.22, 3.00)	1.16 (0.85, 1.46)	2.50 (1.64, 3.69)	*Z* = –10.412	< 0.001
LDL-C, M (Q*1*, Q*3*)	3.20 (2.71, 3.77)	2.98 (2.64, 3.67)	3.27 (2.79, 3.77)	*Z* = –1.743	0.081

The data are presented as the means ± SDs or medians (interquartile ranges). Group comparisons were made via independent *t*-tests for normally distributed data, Mann–Whitney U tests for non-normally distributed data, and chi–square tests for categorical variables. *p* < 0.05 was considered statistically significant. NC, normal control; MAFLD, metabolic dysfunction-associated fatty liver disease; MCH, melanin-concentrating hormone; SBP, systolic blood pressure; DBP, diastolic blood pressure; ALT, alanine aminotransferase; AST, aspartate aminotransferase; TC, total cholesterol; TG, triglycerides; HDL-C, high-density lipoprotein cholesterol; LDL-C, low-density lipoprotein cholesterol; FPG, fasting plasma glucose; HOMA-IR, homeostatic model assessment for insulin resistance; BMI, body mass index; TyG index, triglyceride–glucose index.

The dataset (*n* = 319) was randomly divided into a training set and a test set at an 8:2 ratio. A total of 255 participants were assigned to the training set, and 64 participants were assigned to the test set. There were no significant differences in baseline characteristics between the training and test sets (Supplementary Table 3, see Supplementary material associated with this article online).

### Selection of variables

**FIGURE 1:**
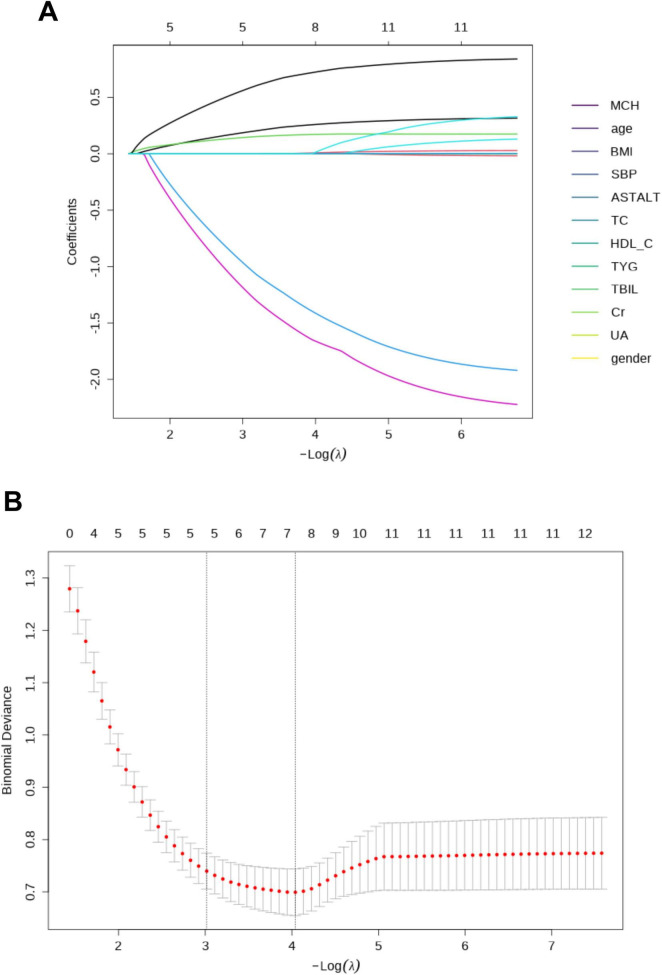
The least absolute shrinkage and selection operator (LASSO) regression model was employed to identify factors associated with MAFLD patients. **(A)** The optimal λ selection in the LASSO model was determined using 10-fold cross-validation, guided by the minimum criteria (the left dotted vertical line) and the 1-SE criteria (the right dotted vertical line). **(B)** The LASSO coefficient profiles for all 13 features revealed that five features with non-zero coefficients were selected at the optimal λ.

The optimal tuning parameter (lambda) in the LASSO model was selected using 10-fold cross-validation. Dotted vertical lines were drawn at the optimal lambda values based on the minimum criterion and the 1-standard error (1-SE) criterion. At the optimal lambda value, five variables with non-zero coefficients were identified. To assess multicollinearity among the selected predictors, the variance inflation factor (VIF) was calculated. Variables with evidence of multicollinearity (VIF > 10) were excluded. After this process, the final predictive variables included MCH, the TyG index, BMI, AST/ALT, and HDL-C. The coefficients for each selected variable are shown (Figure 1).

### Model construction and validation

**FIGURE 2:**
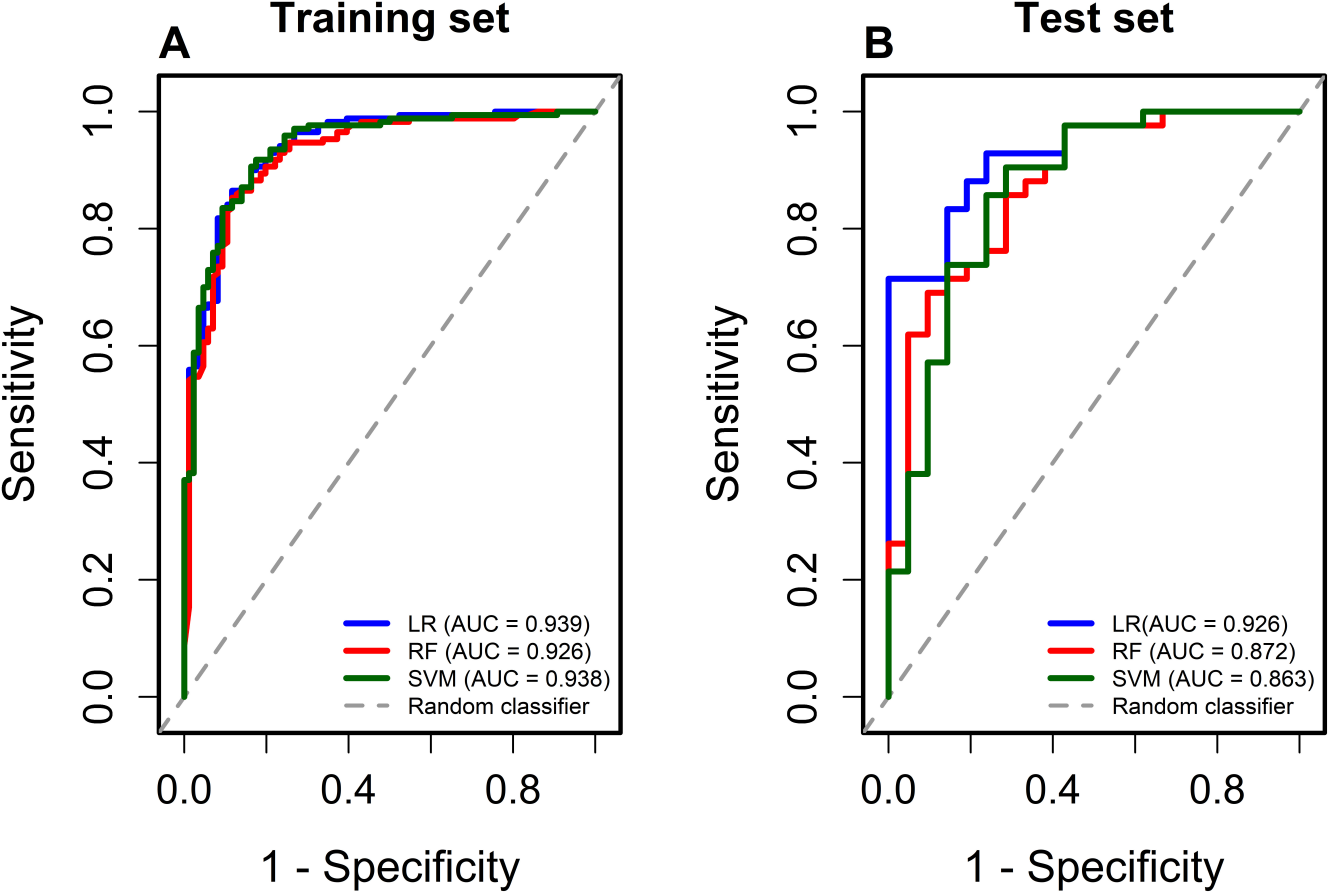
ROC of models in training dataset and testing dataset. **(A)** Represented training dataset. **(B)** Represented testing dataset.

**FIGURE 3:**
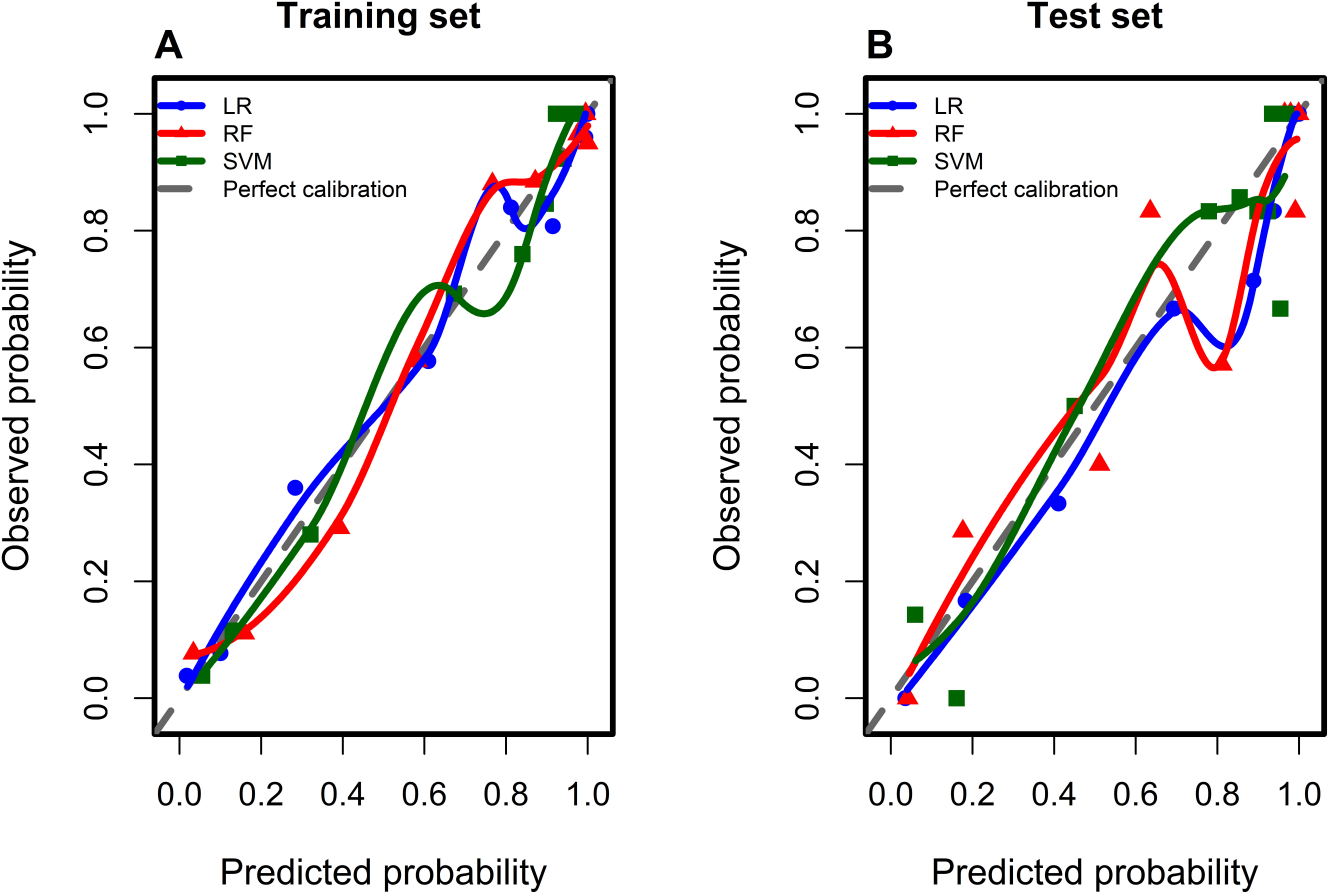
Calibration plot in training dataset and testing dataset. **(A)** Represented training dataset. **(B)** Represented testing dataset.

Using the five variables identified through LASSO regression, three machine learning algorithms—Logistic Regression (LR), Random Forest (RF), Support Vector Machine Model (SVM)—were employed to develop predictive models for the training set. A comprehensive comparison of model performance in the training and test datasets is presented in Figure 2 and Tables 2, 3. The LR model demonstrated favorable predictive performance in the training set, with an AUC of 0.939 (95% CI 0.884–0.968), and achieved an AUC of 0.926 (95% CI 0.865–0.957) in the test set. Consistent with its discrimination performance, the LR model also achieved lower Brier scores, indicating better overall prediction accuracy compared with the other models. Calibration plots of the four models in the training and test datasets are shown in Figure 3. The 45° line between the *X*-axis and the *Y*-axis indicates good agreement between predicted and observed outcomes. Calibration plots of three models in the training dataset and testing dataset are shown in Figure 3. The curve at 45° between the *X*−axis and the *Y*−axis indicates good consistency of the model. Based on these results, the LR model was selected as the final predictive model. Furthermore, bootstrap resampling with 1,000 iterations was conducted in the training set, yielding a mean AUC of 0.937 and an optimism value of 0.0072, indicating that the LR model demonstrated good stability and robustness. The results of the bootstrap resampling are displayed in Supplementary Figure 2.

**TABLE 2:** Performance of models on training set (five-fold CV).

Model	Accuracy	Sensitivity	Specificity	F1	Brier	AUC
LR	0.879 (0.849-0.918)	0.929 (0.884-0.960)	0.779 (0.702-0.874)	0.911	0.088 (0.064-0.113)	0.939 (0.884-0.968
RF	0.858 (0.832-0.898)	0.929 (0.895-0.934)	0.779 (0,712-0.857)	0.911	0.099 (0.086-0.106)	0.922 (0.883-0.952)
SVM	0.886 (0.859-0.938)	0.941 (0.914-0.977)	0.756 (0.714-0.885)	0.910	0.092 (0.065-0.106)	0.908 (0.861-0.952)

AUC, Area Under the Roc Curve; F1, F1 score; Brie, Brier score; LR, Logistic Regression; RF, Random Forest; SVM, support vector machine model. Accuracy = (TP + TN)/(TP + TN + FP + FN). Sensitivity = TP/(TP + FN) Specificity = TN/(TN + FP). F1 score = 2/([1/Recall] + [1/Precision]). FN, false negatives; FP, false positives; TN, true negatives; TP, true positives.

**TABLE 3:** Performance of models on testing tset.

Model	Accuracy	Sensitivity	Specificity	F1	Brier	AUC
LR	0.857 (0.730-0.820)	0.929 (0.872-1.000)	0.714 (0.5710.789)	0.897	0.108 (0.081-0.207)	0.926 (0.865-0.957)
RF	0.794 (0.667-0.873)	0.905 (0.867-0.933)	0.571 (0.408-0.650)	0.854	0.127 (0.087-0.146)	0.891 (0.818-0.976)
SVM	0.810 (0.749-0.866)	0.905 (0.842-0.955)	0.619 (0.548-0.778)	0.864	0.134 (0.084-0.217	0.832 (0.719-0.956)

AUC, Area Under the Roc Curve; F1, F1 score; Brie, Brier score; LR, Logistic Regression; RF, Random Forest; SVM, support vector machine model. Accuracy = (TP + TN)/(TP + TN + FP + FN). Sensitivity = TP/(TP + FN) Specificity = TN/(TN + FP). F1 score = 2/([1/Recall] + [1/Precision]). FN, false negatives; FP, false positives; TN, true negatives; TP, true positives.

**FIGURE 4:**
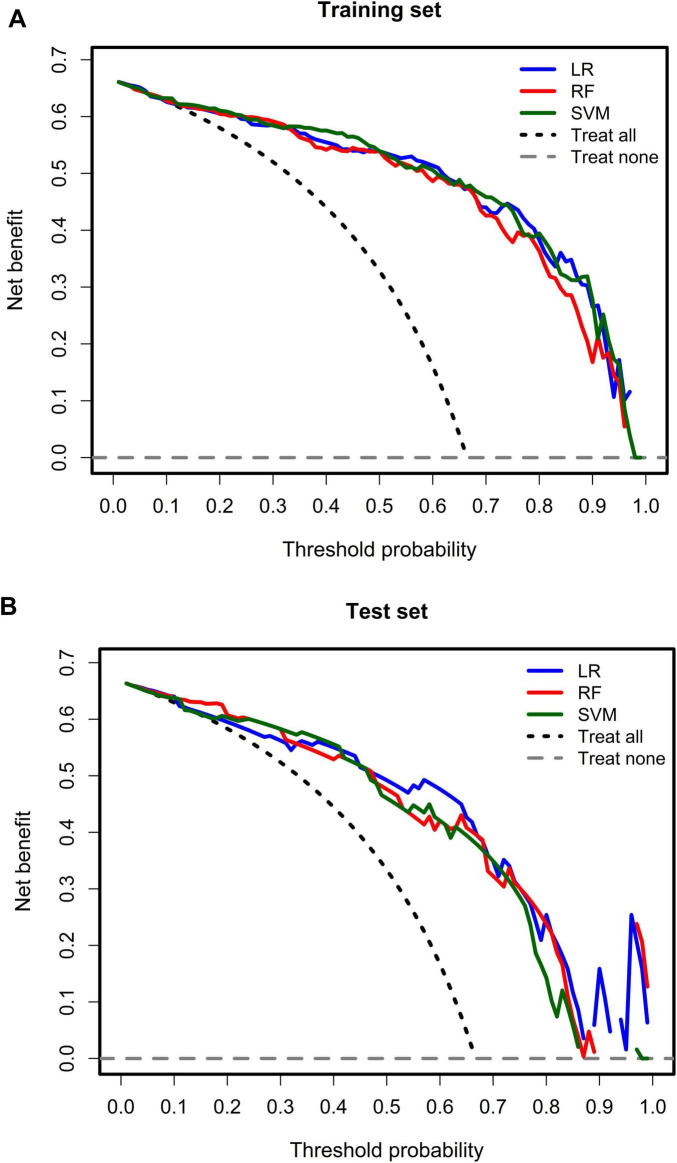
Decision curves for training dataset and testing dataset. **(A)** Represented training dataset. **(B)** Represented testing dataset.

The decision curve analysis showed that all three models yielded a positive net benefit for predicting MAFLD across a range of clinically relevant threshold probabilities in both the training and test datasets, compared with the default strategies of treating all or treating none. Among the models, the LR model consistently demonstrated the highest net benefit across most threshold ranges, indicating good clinical usefulness for decision-making (see Figure 4).

**FIGURE 5:**
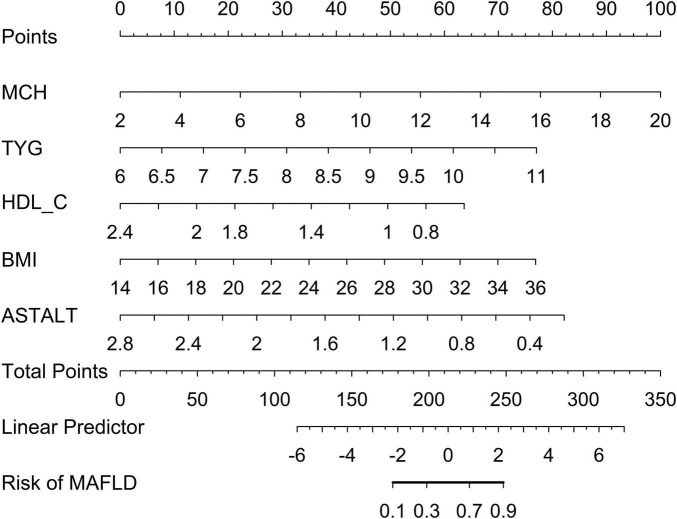
Nomogram for estimation of MAFLD.

Therefore, the LR model was subsequently converted into a nomogram to facilitate its interpretation and clinical application (Figure 5). Each factor in the nomogram was assigned an individual score based on its value, and a total score was calculated by summing the scores of all factors. The final score derived from the nomogram could be utilized to estimate the risk of MAFLD.

Multivariable logistic regression analysis showed that higher levels of MCH (OR = 1.35, 95% CI: 1.17–1.59), TyG index (OR = 2.43, 95% CI: 1.42–4.26), and BMI (OR = 1.38, 95% CI: 1.17–1.16) were independently associated with higher odds of MAFLD. In contrast, higher HDL-C levels (OR = 0.18, 95% CI: 0.04–0.72) and a higher AST/ALT ratio (OR = 0.12, 95% CI: 0.04–0.36) were independently associated with lower odds of MAFLD (Table 4).

**TABLE 4:** Multivariable logistic regression analysis for MAFLD in the training cohort.

Variable	β	OR (95% CI)	*P*-value
MCH	0.3007688	1.35 (1.17–1.59)	< 0.001*
TyG	0.8877737	2.43 (1.42–4.26)	0.001*
HDL_C	-1.7402024	0.18 (0.04–0.72)	0.019*
AST/ALT	-2.1409328	0.12 (0.04–0.36)	<0.001*
BMI	0.3215397	1.38 (1.17–1.66)	<0.001*

*p < 0.05.

### Multivariate logistic regression analysis of the associations between MCH and MAFLD in subgroups stratified by sex, age, and BMI

To explore the relationship between MCH and MAFLD, we conducted multivariate logistic regression analyses stratified by sex, age (≤ 40 vs. > 40 years), and BMI. For BMI stratification, we used the World Health Organization (WHO) recommended cutoff for Asian populations (30), defining normal weight as < 24 kg/m^2^ and overweight/obesity as ≥ 24 kg/m^2^. The positive association between MCH and MAFLD was consistent and significant across all subgroups. Higher MCH was a significant risk factor for MAFLD in both males (OR = 1.91) and females (OR = 1.39), as well as in both younger (OR = 1.92) and older (OR = 1.38) participants (all *P* < 0.001). This association also remained significant in both BMI strata. Crucially, no significant interaction was detected between MCH and sex (P for interaction = 0.359) or BMI (P for interaction = 0.334), indicating the robustness of the association across these groups (Table 5 and Supplementary Figure 3, see the Supplementary material associated with this article online.).

**TABLE 5:** Associations between MCH and MAFLD by sex, BMI, and age.

Variables	n (%)	OR (95%CI)	*P*	P for interaction
Gender				0.359
Male	174 (54.55)	1.91 (1.32∼2.77)	**< 0.001**	
Female	145 (45.45)	1.39 (1.17∼1.66)	**<0.001**	
Age				0.194
< 40	131 (41.07)	1.92 (1.31∼2.81)	**< 0.001**	
> 40	188 (58.93)	1.38 (1.15∼1.66)	**<0.001**	
BMI				0.334
Under/normal weigh	125 (39.18)	1.70 (1.30∼2.23)	**< 0.001**	
Overweight/obese	194 (60.82)	1.31 (1.06∼1.62)	**0.011**	

Adjusted for SBP, DBP, TYG index, FPG, ALT, AST, TG, HDL-C. Bold values indicate statistical significance.

### Mediation analysis of the TyG index in the association between MCHandMAFLD

Considering the biological plausibility that MCH, a neuropeptide that regulates energy metabolism, is associated with insulin resistance, we proceeded to investigate whether the triglyceride–glucose (TyG) index mediates the association between MCH and MAFLD. After adjustment for age, sex, blood pressure, and BMI, the mediation analysis demonstrated a statistically significant indirect association between MCH and MAFLD through the TyG index (indirect effect = 0.003; 95% bootstrap CI: 0.0005–0.01). The TyG index accounted for 10.89% of the total association between MCH and MAFLD, suggesting that this association may be partly explained by differences in the TyG index (Supplementary Table 4 and Supplementary Figure 4, see Supplementary material associated with this article online).

### Interaction analysis of MCH and the TyG index

To further investigate the potential interaction between MCH and the TyG index on MAFLD, we included an interaction term (MCH × TyG) in a binary logistic regression model adjusted for relevant confounders. The analysis revealed that the interaction term was not statistically significant (OR = 1.002, 95% CI: 0.895–1.21, *p* = 0.797). These findings indicate that the TyG index did not significantly modify the association between MCH and MAFLD. In other words, the association of MCH with the risk of MAFLD was consistent across different TyG index levels, and no synergistic or antagonistic effects.

## Discussion

In this study, we conducted a retrospective analysis of the clinical characteristics of MAFLD. Using LASSO regression, factors including MCH, the TyG index, BMI, HDL-C, and AST/ALT were selected to develop three predictive models. Model validation demonstrated that logistic regression exhibited robust discriminative performance and clinical utility. Furthermore, a nomogram was constructed to visualize the predictive model. We also observed that both the melanin-concentrating hormone (MCH) index and the triglyceride glucose (TyG) index were independently associated with MAFLD. Suggesting a potential link between central regulation, peripheral insulin resistance, and hepatic pathology.

MAFLD has become a major global public health challenge, with steatosis as its most prominent feature. A core pathophysiological mechanism involves a vicious cycle between IR and hepatic fat accumulation (31). Early IR increases adipose tissue lipolysis, leading to elevated circulating free fatty acids. The accumulation of fatty acids exacerbates IR and hyperinsulinemia (32), leading to further lipid accumulation in hepatocytes. This vicious cycle promotes MAFLD development and may further triggering the activation of hepatic stellate cells (33). Hyperinsulinemia and free fatty acids disrupt cellular signaling, causing endoplasmic reticulum (ER) stress, impairing autophagy, and promoting metabolic inflammation, thereby aggravating hepatocyte injury and fibrotic progression. As a neuropeptide secreted by the hypothalamus, numerous studies have shown that MCH may be a crucial system for controlling the process of food intake. In 1996, intracerebroventricular (ICV) injection of MCH in rats increased food intake by 150–200% (34). MCH receptor 1 (MCH-R1) deficiency (35) leads to weight loss in both lean and obese mice, reduces food intake, increases locomotor activity, and increases energy expenditure (36). Previous animal experiments have shown that MCH can downregulate the phosphorylation of insulin receptor substrate (IRS-1) in the liver, thereby weakening the physiological effects of insulin, and upregulate the expression of sterol regulatory element-binding protein-1c (SREBP-1c), promoting intrahepatic triglyceride synthesis and accumulation (37). Central infusion of MCH in mice, mediated through the SIRT1/FoxO1 pathway in the arcuate nucleus, leads to increased food intake, obesity, glucose intolerance, and increased hepatic TG content (38). Chronic intracerebroventricular infusion of an MCH-R1 antagonist significantly improved the obese phenotype, including hyperinsulinemia, and markedly reduced the hepatic TG content, thereby ameliorating steatohepatitis (39). In addition to stimulating food intake via homeostatic and hedonic pathways, MCH reduces energy expenditure and locomotor activity and induces adiposity, weight gain, glucose intolerance and peripheral lipid storage (21).

Circulating MCH may be secreted from hypothalamic neuronal terminals and subsequently enter the systemic circulation by crossing the blood–brain barrier. In addition, previous studies have reported that endocrine tissues such as the pancreas may also produce a proportion of MCH. However, the peripheral sources of MCH remain largely unclear. Moreover, earlier research has shown that plasma MCH concentrations decrease in patients with improved glucose tolerance (34). Taken together, these findings suggest a close association between MCH and insulin resistance, obesity, and related metabolic disturbances. Owing to its simplicity and non-invasiveness, the TyG index has become one of the most widely used surrogate markers for IR in recent years (40, 41). A study involving 809 participants in the United States revealed that the TyG index and TyG-related indices are highly important for identifying MAFLD risk (42). Although the triglyceride–glucose (TyG) index is not a liver-specific biomarker, it is a well-established surrogate indicator of systemic insulin resistance. Insulin resistance plays a central role in the pathogenesis of MAFLD, particularly through its effects on hepatic glucose and lipid metabolism. Hepatic insulin resistance promotes increased hepatic *de novo* lipogenesis, impaired suppression of gluconeogenesis, and lipid accumulation within hepatocytes. In this context, the TyG index may serve as an indirect but clinically relevant marker linking peripheral insulin resistance to hepatic metabolic. In our study, we established a three mainstream machine learning models and incorporated five variables into its construction: MCH, TyG index, AST/ALT, BMI and HDL-C. The optimal predictive model was represented as a nomogram. The LR model performed well, with AUCs of 0.939 and 0.926 in the training and testing datasets, respectively. Model calibration was quantitatively assessed using Brier scores and the LR model showed good agreement between predicted and the observed outcome. Decision curve analysis (DCA) showed that the LR model provided the highest net benefit across most threshold ranges, suggesting good clinical utility. Finally, the five variables were included in the multivariate logistic regression analysis. We found that MCH and TyG index were independent risk factors for MAFLD. Interestingly, logistic regression outperformed the more complex machine learning models in this study. This finding may be attributed to several factors. First, the predictors included in the final model were limited in number and demonstrated relatively linear and stable associations with MAFLD, which favors the performance of traditional statistical models. Second, the moderate sample size may have constrained the ability of more complex algorithms to fully leverage their advantages, potentially increasing the risk of overfitting despite internal validation. Importantly, the application of machine learning in this study was not intended to replace conventional regression models but rather to support feature selection, model comparison, and robustness assessment. The comparable or inferior performance of complex models highlights that increased algorithmic complexity does not necessarily translate into superior predictive performance in all clinical settings. From a clinical perspective, the superior performance and interpretability of logistic regression further enhance its applicability as a screening tool. For the application of this model, the total score obtained by summing the points assigned to each variable in the nomogram can be further converted into an estimated probability of MAFLD. A higher total score corresponds to a greater predicted risk of developing MAFLD. Based on this predictive model, the nomogram may serve as a practical screening tool to identify individuals at high risk of MAFLD, thereby facilitating early risk stratification and the implementation of targeted preventive interventions.

The mediation analysis indicated that the TyG index statistically accounted for approximately 11% of the association between MCH and MAFLD. Although this proportion may appear modest in magnitude, it should be interpreted in the context of a modest mediation effect. In complex metabolic diseases such as MAFLD, multiple biological pathways often act in parallel, and individual mediators may each explain only a small proportion of the overall association. Even a relatively small mediation proportion may therefore reflect a meaningful biological link, particularly when the mediator represents a well-established metabolic pathway. Given the cross-sectional design, the mediation results should be interpreted as exploratory and hypothesis-generating, and future longitudinal studies are needed to clarify the relative contributions of multiple pathways linking MCH and MAFLD. Several alternative and complementary mechanisms may also underlie the association between MCH and MAFLD. Experimental evidence suggests that MCH neurons are modulated by insulin, and that chronic central administration of MCH can induce insulin resistance through mechanisms independent of weight gain (23). In addition, MCH has been shown to directly upregulate hepatic sterol regulatory element–binding protein 1c (SREBP-1c), thereby promoting *de novo* lipogenesis, which represents another important pathogenic pathway. Moreover, the central effects of MCH in increasing food intake may directly contribute to energy surplus and hepatic fat accumulation, without fully relying on insulin resistance as an intermediary. At the same time, the TyG index, as a surrogate marker of insulin resistance, primarily reflects systemic insulin sensitivity, and its ability to capture liver-specific insulin resistance may be limited. As a result, the magnitude of the indirect effect observed in this analysis may underestimate the true mediating role of insulin resistance in the pathological process linking MCH to MAFLD.

We further explore the interplay between MCH and the TyG index in the pathogensis of MAFLD, The results revealed that the no statistically significant interaction was observed between MCH and the TyG index in relation to MAFLD. This finding suggests that the association between MCH and MAFLD does not materially differ across levels of TyG. From a biological perspective, the absence of interaction may indicate that MCH-related pathways and insulin resistance–related metabolic alterations act in a largely additive rather than synergistic manner. In other words, while the TyG index may partially account for the association between MCH and MAFLD, the effect of MCH appears to be relatively consistent across different degrees of insulin resistance. Clinically, this finding implies that the association between MCH and MAFLD is not confined to specific metabolic subgroups defined by TyG levels, which may enhance the generalizability of the observed relationship. Previous studies have reported sex differences in the effects of MCH in promoting appetite and reproduction (43, 44). Therefore, this study conducted further subgroup analyses stratified by sex, age, and BMI. The subgroup analyses supported the robustness of the association between MCH and MAFLD. The association remained significant in both male and female populations, as well as in younger (≤ 40 years) and older (> 40 years) participants, with no significant interactions detected. Notably, in non-obese participants (BMI < 24 kg/m^2^), the association remained significant, providing new insights into the pathogenesis of non-obese MAFLD. Specifically, MCH-mediated insulin resistance and dysregulated lipid metabolism may represent a pathogenic pathway independent of obesity. These subgroup findings may be limited by sample size and warrant validation in larger studies. Although this study yielded several important findings, it still has several limitations. First, this was a single-center, small-sample, cross-sectional study, which cannot establish a causal relationship between MCH, the TyG index, and MAFLD; the relatively small sample size of patients used to develop a new prediction model might be associated with selection bias, reduce the statistical power, increase the risk of type II errors, It may also compromise model accuracy. Second, the diagnosis of MAFLD was based on abdominal ultrasound rather than liver biopsy or advanced imaging modalities. Although ultrasound is widely used in large-scale epidemiological studies due to its feasibility and non-invasiveness, it has limited sensitivity for mild steatosis and cannot reliably distinguish simple steatosis from steatohepatitis. Consequently, misclassification bias cannot be excluded, which may have influenced the observed associations and the evaluation of disease severity. Third, the model was built for a population at high risk of MAFLD. It cannot, however, be generally applicable or applied to a larger population. Fourth, the predictive model was validated exclusively using internal validation methods, including a train–test split and cross-validation. The absence of external validation represents a major limitation, as it restricts the assessment of the model’s robustness and applicability across different populations and clinical settings. Future studies with independent external cohorts are required to confirm the generalizability and clinical utility of this nomogram.

## Conclusion

Both serum MCH and the TyG index were found to be independently associated with MAFLD in this cross-sectional cohort. A logistic regression–based predictive model was developed to estimate MAFLD risk. In appropriate clinical or nutritional settings, the model can be used as a preliminary decision- support tool to assist clinicians or dietitians in prioritizing patients for additional diagnostic evaluation, closer monitoring, or targeted nutritional management. For example, in routine clinical assessments or nutritional screening workflows, the model may help distinguish higher-risk individuals from those at lower risk, thereby supporting more efficient allocation of clinical resources. However, the clinical utility and applicability of this model still require validation in larger-scale prospective studies.

## Data Availability

The data that support the findings of this study are available from the corresponding author upon reasonable request.

## References

[1] Eslam M Sanyal A George J. International consensus panel. A new definition for metabolic dysfunction-associated fatty liver disease: an international expert consensus statement. *J Hepatol.* (2020) 73:202–9. 10.1016/j.jhep.2020.07.045 32278004

[2] Long M Noureddin M Lim JK. AGA clinical practice update on diagnosis and management of nonalcoholic fatty liver disease in lean individuals:expert review. *Gastroenterology.* (2022) 163:764–74.e1. 10.1053/j.gastro.2022.04.038 35842345 PMC9398982

[3] Powell E Wong V Rinella M. Nonalcoholic fatty liver disease. *Lancet.* (2021) 397:2212–24. 10.1016/S0140-6736(21)00712-X 33894145

[4] Beygi M Ahi S Zolghadri S Stanek A. Management of metabolic-associated fatty liver disease/metabolic dysfunction-associated steatotic liver disease: from medication therapy to nutritional interventions. *Nutrients.* (2024) 16:2142. 10.3390/nu16142142 39064665 PMC11279539

[5] Zhang W Cheng Q Yin L Liu Y Chen L Jiang Z et al. Jujuboside A through YY1/CYP2E1 signaling alleviated type 2 diabetes-associated fatty liver disease by ameliorating hepatic lipid accumulation, inflammation, and oxidative stress. *Chem Biol Interact.* (2024) 400:111157. 10.1016/j.cbi.2024.111157 39059604

[6] Zhao L Zeng Q Zhou X Tang L Wang Y Han Q et al. Impact of nonalcoholic fatty liver disease and fibrosis on mortality and kidney outcomes in patients with type 2 diabetes and chronic kidney disease: a multicohort longitudinal study. *Diabetes Obes Metab.* (2024) 26:4241–50. 10.1111/dom.15694 39021330

[7] Singh S Allen A Wang Z Prokop L Murad M Loomba R. Fibrosis progression in nonalcoholic fatty liver vs nonalcoholic steatohepatitis: a systematic review and meta-analysis of paired-biopsy studies. *Clin Gastroenterol Hepatol.* (2015) 13:643–54.e1-9. 10.1016/j.cgh.2014.09.035 24768810 PMC4208976

[8] Berzigotti A. Getting closer to a point-of-care diagnostic assessment in patients with chronic liver disease: controlled attenuation parameter for steatosis. *J Hepatol.* (2014) 60:910–2. 10.1016/j.jhep.2014.01.018 24486330

[9] Sasso M Miette V Sandrin L Beaugrand M. The controlled attenuation parameter (CAP): a novel tool for the noninvasive evaluation of steatosis using Fibroscan^®^. *Clin Res Hepatol Gastroenterol.* (2012) 36:13–20. 10.1016/j.clinre.2011.08.001 21920839

[10] Karlas T Petroff D Sasso M Fan J Mi Y de Lédinghen V et al. Individual patient data meta-analysis of controlled attenuation parameter (CAP) technology for assessing steatosis. *J Hepatol.* (2017) 66:1022–30. 10.1016/j.jhep.2016.12.022 28039099

[11] Lédinghen V Vergniol J Capdepont M Chermak F Hiriart J Cassinotto C et al. Controlled attenuation parameter (CAP) for the diagnosis of steatosis: a prospective study of 5323 examinations. *J Hepatol.* (2014) 60:1026–31. 10.1016/j.jhep.2013.12.018 24378529

[12] Zhang S Du T Zhang J Lu H Lin X Xie J et al. The triglyceride and glucose index (TyG) is an effective biomarker to identify nonalcoholic fatty liver disease. *Lipids Health Dis.* (2017) 16:15. 10.1186/s12944-017-0409-6 28103934 PMC5248473

[13] Abbasi F Reaven G. Comparison of two methods using plasma triglyceride concentration as a surrogate estimate of insulin action in nondiabetic subjects: triglycerides × glucose versus triglyceride/high-density lipoprotein cholesterol. *Metabolism.* (2011) 60:1673–6. 10.1016/j.metabol.2011.04.004 21632070

[14] Ling Q Chen J Liu X Xu Y Ma J Yu P et al. The triglyceride and glucose index and risk of nonalcoholic fatty liver disease: a dose–response meta-analysis. *Front Endocrinol.* (2022) 13:1043169. 10.3389/fendo.2022.1043169 36743937 PMC9892833

[15] Guo W Lu J Qin P Li X Zhu W Wu J et al. The triglyceride-glucose index is associated with the severity of hepatic steatosis and the presence of liver fibrosis in nonalcoholic fatty liver disease: a cross-sectional study in Chinese adults. *Lipids Health Dis.* (2020) 19:218. 10.1186/s12944-020-01394-z 33028338 PMC7541277

[16] Simental-Mendía L Rodríguez-Morán M Guerrero-Romero F. The product of fasting glucose and triglycerides as surrogate for identifying insulin resistance in apparently healthy subjects. *Metab Syndr Relat Disord.* (2008) 6:299–304. 10.1089/met.2008.0034 19067533

[17] Vaughan J Fischer W Hoeger C Rivier J Vale W. Characterization of melanin-concentrating hormone from rat hypothalamus. *Endocrinology.* (1989) 125:1660–5. 10.1210/endo-125-3-1660 2759038

[18] Bittencourt J. Anatomical organization of the melanin-concentrating hormone peptide family in the mammalian brain. *Gen Comp Endocrinol.* (2011) 172:185–97. 10.1016/j.ygcen.2011.02.009 21463631

[19] Izawa S Chowdhury S Miyazaki T Mukai Y Ono D Inoue R et al. REM sleep-active MCH neurons are involved in forgetting hippocampus-dependent memories. *Science.* (2019) 365:1308–13. 10.1126/science.aax9238 31604241 PMC7378274

[20] Santollo J Eckel L. The orexigenic effect of melanin-concentrating hormone (MCH) is influenced by sex and stage of the estrous cycle. *Physiol Behav.* (2008) 93:842–50. 10.1016/j.physbeh.2008.01.003 18191424 PMC2573992

[21] Al-Massadi O Dieguez C Schneeberger M López M Schwaninger M Prevot V et al. Multifaceted actions of melanin-concentrating hormone on mammalian energy homeostasis. *Nat Rev Endocrinol.* (2021) 17:745–55. 10.1038/s41574-021-00559-1 34608277

[22] Ludwig D Tritos N Mastaitis J Kulkarni R Kokkotou E Elmquist J et al. Melanin-concentrating hormone overexpression in transgenic mice leads to obesity and insulin resistance. *J Clin Invest.* (2001) 107:379–86. 10.1172/JCI10700 11160162 PMC199192

[23] Pereira-da-Silva M De Souza C Gasparetti A Saad M Velloso L. Melanin-concentrating hormone induces insulin resistance through a mechanism independent of body weight gain. *J Endocrinol.* (2005) 186:193–201. 10.1677/joe.1.06103 16002548

[24] Imbernon M Beiroa D Vázquez M Morgan D Veyrat-Durebex C Porteiro B et al. Central melanin-concentrating hormone influences liver and adipose metabolism via specific hypothalamic nuclei and efferent autonomic/JNK1 pathways. *Gastroenterology.* (2013) 144:636–49.e2. 10.1053/j.gastro.2012.11.030 23142626 PMC3663042

[25] Rajkomar A Dean J Kohane I. Machine learning in medicine. *N Engl J Med.* (2019) 380:1347–58. 10.1056/NEJMra1814259 30943338

[26] Greener J Kandathil S Moffat L Jones DT. A guide to machine learning for biologists. *Nat Rev Mol Cell Biol.* (2022) 23:40–55. 10.1038/s41580-021-00407-0 34518686

[27] Guerrero-Romero F Simental-Mendía L González-Ortiz M Martínez-Abundis E Ramos-Zavala M Hernández-González S et al. The product of triglycerides and glucose, a simple measure of insulin sensitivity. Comparison with the euglycemic-hyperinsulinemic clamp. *J Clin Endocrinol Metab.* (2010) 95:3347–51. 10.1210/jc.2010-0288 20484475

[28] Luo Y Ding W Yang X Zhang Y Li H Wang M et al. Construction and validation of a predictive model for meningoencephalitis in pediatric scrub typhus based on machine learning algorithms. *Emerg Microbes Infect.* (2025) 14:2469651. 10.1080/22221751.2025.2469651 39964062 PMC11892057

[29] Hu X Liu H Zhao X Li Y Zhang H Wang J et al. Automated machine learning-based model predicts postoperative delirium using readily extractable perioperative collected electronic data. *CNS Neurosci Ther.* (2022) 28:608–18. 10.1111/cns.13758 34792857 PMC8928919

[30] Expert Consultation W. Appropriate body-mass index for Asian populations and its implications for policy and intervention strategies. *Lancet.* (2004) 363:157–63. 10.1016/S0140-6736(03)15268-3 14726171

[31] Angulo P Machado M Diehl A. Fibrosis in nonalcoholic fatty liver disease: mechanisms and clinical implications. *Semin Liver Dis.* (2015) 35:132–45. 10.1055/s-0035-1550058 25974899

[32] Tanase D Gosav E Costea C Ciocoiu M Lacatusu C Maranduca M et al. The intricate relationship between type 2 diabetes mellitus (T2DM), insulin resistance (IR), and nonalcoholic fatty liver disease (NAFLD). *J Diabetes Res.* (2020) 2020:3920196. 10.1155/2020/3920196 32832560 PMC7424491

[33] Ioannou G. The role of cholesterol in the pathogenesis of NASH. *Trends Endocrinol Metab.* (2016) 27:84–95. 10.1016/j.tem.2015.11.008 26703097

[34] Qu D Ludwig D Gammeltoft S Piper M Pelleymounter M Cullen M et al. A role for melanin-concentrating hormone in the central regulation of feeding behavior. *Nature.* (1996) 380:243–7. 10.1038/380243a0 8637571

[35] Marsh D Weingarth D Novi D Chen H Trumbauer M Chen A et al. Melanin-concentrating hormone 1 receptor-deficient mice are lean, hyperactive, and hyperphagic and have altered metabolism. *Proc Natl Acad Sci U S A.* (2002) 99:3240–5. 10.1073/pnas.052706899 11867747 PMC122503

[36] Stricker-Krongrad A Dimitrov T Beck B. Central and peripheral dysregulation of melanin-concentrating hormone in obese Zucker rats. *Mol Brain Res.* (2001) 92:43–8. 10.1016/s0169-328x(01)00150-5 11483240

[37] Takahashi K Funahashi H Kayanoki T Taka-Iwa A Ogawa H Ohta M et al. Melanin-concentrating hormone receptor 1 (MCH-R1) signaling in the liver regulates lipid metabolism and insulin sensitivity. *Endocrinology.* (2013) 154:3594–609. 10.1210/en.2013-1090

[38] Al-Massadi O Quiñones M Clasadonte J Nogueiras R Dieguez C Lopez MMCH. regulates SIRT1/FoxO1 and reduces POMC neuronal activity to induce hyperphagia, adiposity, and glucose intolerance. *Diabetes.* (2019) 68:2210–22. 10.2337/db19-0331 31530579 PMC6868473

[39] Ito M Gomori A Suzuki J Sone H Akahoshi S Murai N et al. Antagonism of central melanin-concentrating hormone 1 receptor alleviates steatohepatitis in mice. *J Endocrinol.* (2008) 198:309–15. 10.1677/JOE-08-0110 18523032

[40] DeFronzo R Tobin J Andres R. Glucose clamp technique: a method for quantifying insulin secretion and resistance. *Am J Physiol.* (1979) 237:E214–23. 10.1152/ajpendo.1979.237.3.E214 382871

[41] Vasques A Novaes F de Oliveira Mda S Souza J Yamanaka A Pareja J et al. TyG index performs better than HOMA in a Brazilian population: a hyperglycemic clamp validated study. *Diabetes Res Clin Pract.* (2011) 93:e98–100. 10.1016/j.diabres.2011.05.030 21665314

[42] Wu Z Huang K Bao S Zhang X Li J Kong W et al. The association of triglyceride-glucose-waist circumference with metabolic associated fatty liver disease and the severity of liver steatosis and fibrosis in American adults: a population-based study. *Scand J Gastroenterol.* (2024) 59:561–9. 10.1080/00365521.2023.2289668 38235548

[43] Terrill S Subramanian K Lan R Bowen A Faget V de Lartigue G et al. Nucleus accumbens melanin-concentrating hormone signaling promotes feeding in a sex-specific manner. *Neuropharmacology.* (2020) 178:108270. 10.1016/j.neuropharm.2020.108270 32795460 PMC7544677

[44] Wu M Dumalska I Morozova E van den Pol A Alreja M. Melanin-concentrating hormone directly inhibits GnRH neurons and blocks kisspeptin activation, linking energy balance to reproduction. *Proc Natl Acad Sci U S A.* (2009) 106:17217–22. 10.1073/pnas.0906326106 19805188 PMC2761345

