# Supplementary material for: Combined analysis of the triglyceride–glucose index and melanin-concentrating hormone in metabolic dysfunction–associated fatty liver disease: a machine learning–based study
Source: Front Nutr. 2026 Mar 5;13:1763190. doi: 10.3389/fnut.2026.1763190 (PMC12999439; doi:10.3389/fnut.2026.1763190)

Supplementary Material

# Supplementary Table 1. Hyperparameters of the predictive models

| **Model** | **Hyperparameter** | **Value** |
| --- | --- | --- |
| Logistic regression | Regularization | Ridge (L2) |
|  | Regularization strength (lambda) | 0.0215 |
|  | Solver | glmnet |
|  | Maximum iterations (max_iter) | 1000 |
| Random forest | Number of trees (num.trees) | 500 |
|  | Number of features per split (mtry) | 3 |
|  | Split rule | gini |
|  | min_samples_split(min.node.size) | 5 |
|  | Maximum tree depth (max_depth) | 5 |
| Support vector machine | Kernel | RBF |
|  | Regularization parameter (C) | 2 |
|  | Kernel coefficient (gamma) | 0.1 |

****Supplementary Table 2. Data preprocessing procedures****

| **Step** | **Method** | **Description** |  |
| --- | --- | --- | --- |
| Outlier identification | Interquartile range (IQR) method | Values below Q1 − 1.5×IQR or above Q3 + 1.5×IQR were identified as potential outliers within the training dataset |  |
| Outlier verification | Manual review of original records | All identified outliers were cross-checked against original source records to distinguish data entry errors from true extreme values |  |
| Outlier correction | Data correction | Values resulting from data entry errors were corrected to their accurate values based on original records |  |
| Outlier handling | Winsorization | Observations confirmed as true extreme values were retained and capped at the corresponding IQR-based thresholds |  |
| Missing data imputation | Multiple imputation by chained equations (MICE) with predictive mean matching (PMM) | Five imputed datasets were generated; imputation was performed exclusively within the training dataset to prevent information leakage |  |
| Feature selection | LASSO regression | Feature selection was conducted within the training dataset using five-fold cross-validation |  |
| Feature selection | VIF | (VIF > 10) were excluded |  |

**Supplementary Table 3.** **Demographic and clinical characteristics of participants by the**

**training dataset and the testing dataset**

| **Variables** | **Total (n = 319)** | **test (n = 64)** | **train (n = 255)** | **Statistic** | ***P*** |
| --- | --- | --- | --- | --- | --- |
|  |  |  |  |  |  |
| MCH, Mean ± SD | 11.56 ± 3.05 | 11.32 ± 3.12 | 11.62 ± 3.03 | t=-0.69 | 0.491 |
| Age, Mean ± SD | 44.28 ± 10.97 | 44.36 ± 11.02 | 44.26 ± 10.98 | t=0.06 | 0.950 |
| Height, Mean ± SD | 152.97 ± 40.96 | 154.22 ± 40.59 | 152.65 ± 41.13 | t=0.27 | 0.785 |
| Weight, Mean ± SD | 67.35 ± 11.65 | 68.43 ± 13.12 | 67.08 ± 11.27 | t=0.83 | 0.407 |
| BMI, Mean ± SD | 25.02 ± 3.09 | 25.02 ± 3.15 | 25.02 ± 3.08 | t=0.00 | 0.999 |
| SBP, Mean ± SD | 127.21 ± 17.13 | 130.44 ± 16.68 | 126.40 ± 17.18 | t=1.69 | 0.092 |
| DBP, Mean ± SD | 79.93 ± 12.49 | 81.64 ± 12.54 | 79.51 ± 12.46 | t=1.22 | 0.222 |
| FPG, Mean ± SD | 6.34 ± 2.82 | 6.77 ± 3.28 | 6.24 ± 2.69 | t=1.35 | 0.176 |
| ASTALT, Mean ± SD | 1.11 ± 0.43 | 1.17 ± 0.46 | 1.09 ± 0.42 | t=1.31 | 0.192 |
| TBIL, Mean ± SD | 13.29 ± 6.20 | 12.65 ± 4.93 | 13.45 ± 6.48 | t=-0.93 | 0.353 |
| Cr, Mean ± SD | 76.48 ± 55.43 | 84.17 ± 116.70 | 74.55 ± 21.23 | t=1.24 | 0.215 |
| TC, Mean ± SD | 5.51 ± 1.56 | 5.93 ± 2.57 | 5.41 ± 1.16 | t=1.58 | 0.118 |
| HDL-C, Mean ± SD | 1.25 ± 0.31 | 1.27 ± 0.33 | 1.25 ± 0.30 | t=0.49 | 0.627 |
| TYG, Mean ± SD | 9.00 ± 0.77 | 9.01 ± 0.74 | 9.00 ± 0.78 | t=0.09 | 0.927 |
| ALT, M (Q₁, Q₃) | 26.00 (17.00, 41.00) | 27.00 (18.00, 38.25) | 26.00 (17.00, 41.00) | Z=-0.12 | 0.902 |
| AST, M (Q₁, Q₃) | 27.00 (22.00, 33.00) | 27.00 (23.75, 33.25) | 26.00 (22.00, 33.00) | Z=-0.97 | 0.332 |
| TG, M (Q₁, Q₃) | 1.81 (1.22, 3.00) | 1.92 (1.33, 2.75) | 1.80 (1.20, 3.04) | Z=-0.73 | 0.465 |
| LDL-C, M (Q₁, Q₃) | 3.20 (2.71, 3.77) | 3.24 (2.71, 3.85) | 3.19 (2.72, 3.77) | Z=-0.48 | 0.630 |
| Gender, n(%) |  |  |  | χ²=0.67 | 0.414 |
| female | 145 (45.45) | 32 (50.00) | 113 (44.31) |  |  |
| male | 174 (54.55) | 32 (50.00) | 142 (55.69) |  |  |
|  | | | | | |

**Abbreviations:** NC: normal control; MAFLD: metabolic dysfunction-associated fatty liver disease; MCH: melanin-concentrating hormone; SBP: systolic blood pressure; DBP: diastolic blood pressure; ALT: alanine aminotransferase; AST: aspartate aminotransferase; TC: total cholesterol; TG: triglycerides; HDL-C: high-density lipoprotein cholesterol; LDL-C: low-density lipoprotein cholesterol; FPG: fasting plasma glucose; HOMA-IR: homeostatic model assessment for insulin resistance; BMI: body mass index; TyG index: triglyceride‒glucose index

**Supplementary Table 4.**

| **Effect** | **Estimate** | **Lower Upper (95%CI)** | **P** | **Mediation** |
| --- | --- | --- | --- | --- |
|  |  |  |  |  |
| Indirect  Direct  Total | 0.003  0.026  0.029 | 0.0005 0.01  0.0152 0.03  0.0175 0.04 | **0.012**  **<.001**  **<.001** | 10.32  89.68  100.00 |

Abbreviations: CI, confidence interval.

**Supplementary Figure 1.**Flowchart of the study

**
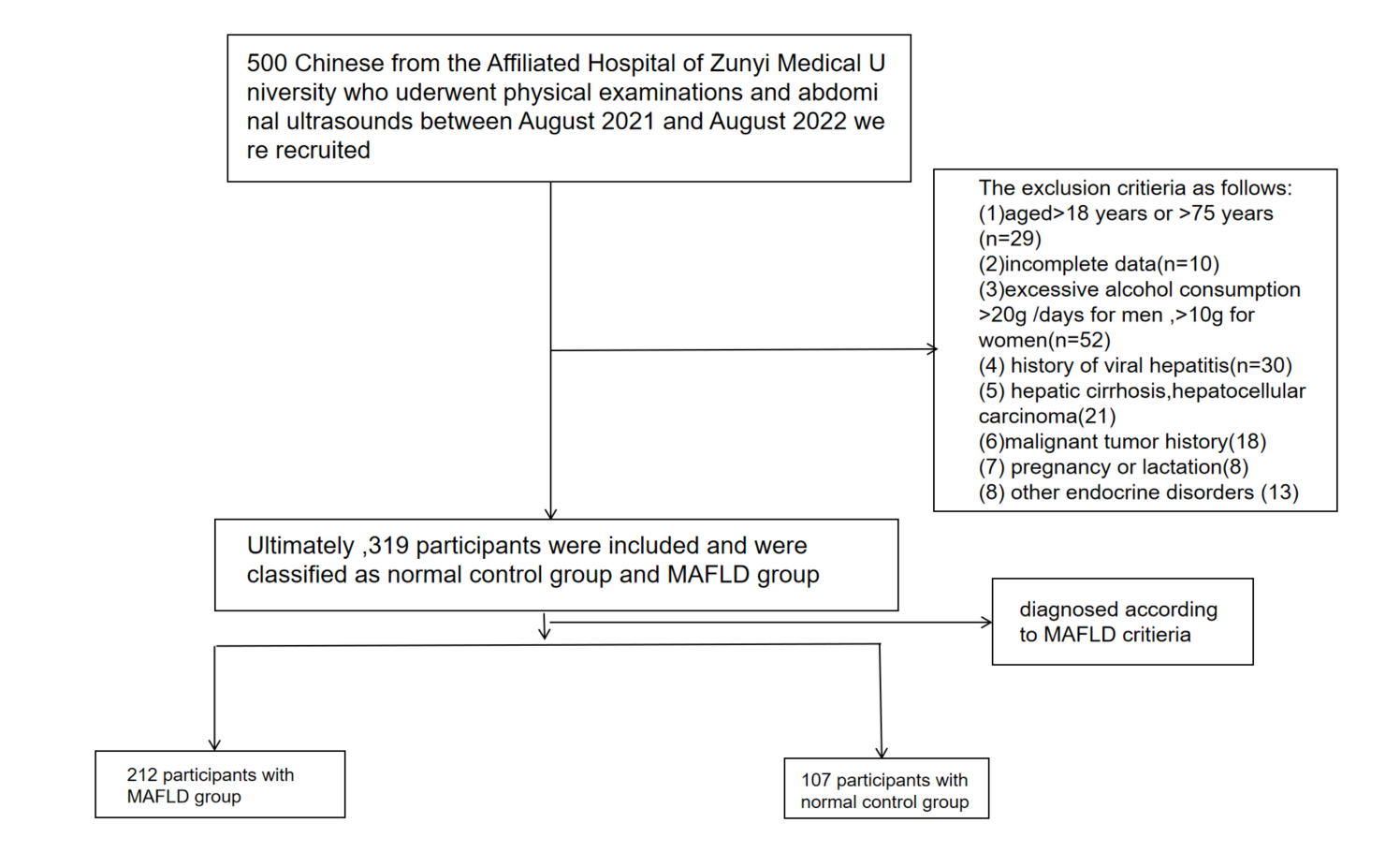
**

**Supplementary Figure 2**





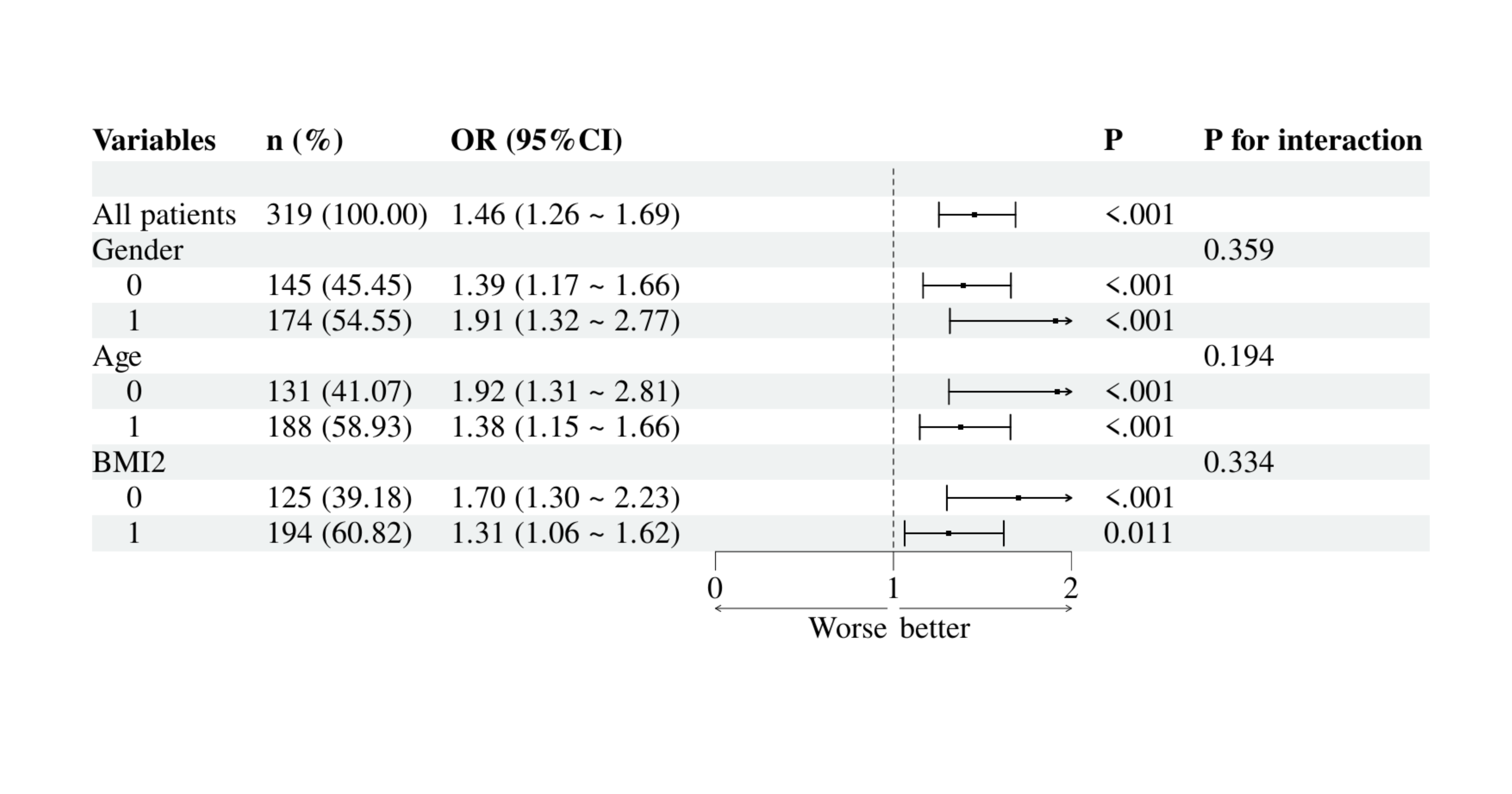
**Supplementary Figure 3.**Subgroup analysis of the between MCH and MAFLD patients (by sex, BMI, and age) — corresponding forest plot: 0 = female, <40 years old, normal weight 1 = male, >40 years old, overweight or obese

**Supplementary Figure 4.**Relationship between MCH, TyG index, and MAFLD.


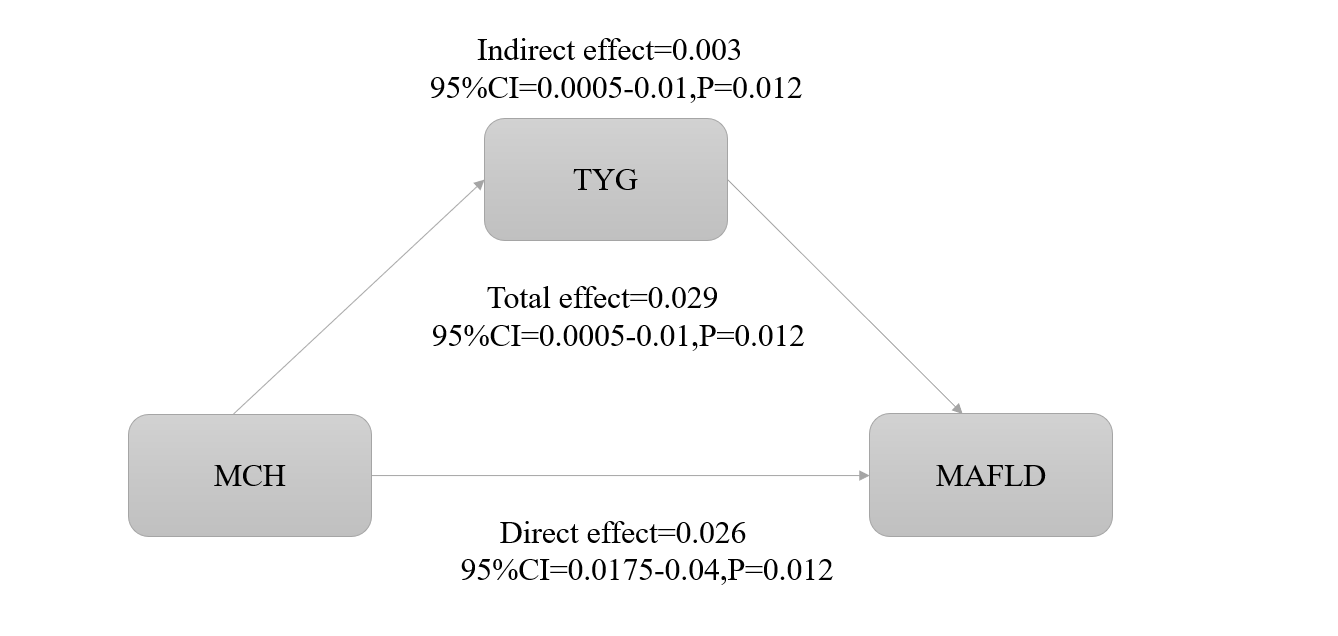

Supplement: Supplementary file 1 [file Table_1.docx]
